# Checklist of bees (Hymenoptera: Apoidea) from managed emergent wetlands in the lower Mississippi Alluvial Valley of Arkansas

**DOI:** 10.3897/BDJ.6.e24071

**Published:** 2018-05-09

**Authors:** Phillip L Stephenson, Terry L Griswold, Michael S Arduser, Ashley P G Dowling, David G Krementz

**Affiliations:** 1 Arkansas Cooperative Fish and Wildlife Research Unit, Department of Biological Sciences, University of Arkansas, Fayetteville, AR, United States of America; 2 USDA ARS Pollinating Insects Research Unit, Utah State University, Logan, UT, United States of America; 3 Missouri Department of Conservation - Retired, Webster Groves, MO, United States of America; 4 Department of Entomology, University of Arkansas, Fayetteville, AR, United States of America; 5 U.S. Geological Survey, Arkansas Cooperative Fish and Wildlife Research Unit, Department of Biological Sciences, University of Arkansas, Fayetteville, AR, United States of America

**Keywords:** Apoidea, Arkansas, bee, biodiversity, emergent wetland, Mississippi Alluvial Valley, native species, state record, range expansion

## Abstract

**Background:**

Here we present the results from a two-year bee survey conducted on 18 managed emergent wetlands in the lower Mississippi Alluvial Valley of Arkansas, USA. Sample methods included pan traps, sweep netting and blue-vane traps. We document 83 bee species and morphospecies in 5 families and 31 genera, of which 37 species represent first published state records for Arkansas. The majority of species were opportunistic wetland species; only a small number were wetland-dependent species or species largely restricted to alluvial plains.

**New information:**

We present new distributional records for bee species not previously recorded in managed emergent wetlands and report specimens of thirty-seven species for which no published Arkansas records exist, expanding the known ranges of *Ceratina cockerelli*, *Diadasia enavata, Lasioglossum creberrimum, Svastra cressonii* and *Dieunomia triangulifera*. We also distinguish opportunistic wetland bee species from wetland-dependent and alluvial plain-restricted species.

## Introduction

Wetlands of one type or another occur throughout North America and, in some parts of the country, dominate the landscape (Mitsch and Gosselink 2015). Wetlands typically have a unique biota, with numerous obligate and opportunistic species (Niering 1988, Kingsbury and Gibson 2012), including various plants (Lichvar et al. 2016) that provide cover and food for many vertebrates such as migratory birds (Kross et al. 2008, Bellrose 1976). A number of these plants are insect-pollinated or experience enhanced reproduction as a consequence of insect visitation (Harder and Barrett 1992, Lippok et al. 2000,Sohmer and Sefton 1978, Loose et al. 2005, Reader 1975,Klips 1999, Estes and Thorp 1974), indicating that pollination services in wetlands are an important part of wetland systems and their function.

While bees are considered the most important pollinators in most North American communities (Michener 2007), relatively little is known about bee faunas occupying or servicing wetland communities in North America. Some wetland communities have been surveyed, amongst these the Florida Everglades (Pascarella et al. 2000), some north-central Florida wetlands (Hall and Ascher 2010), fens in southern Michigan (Fiedler et al. 2011), playa lakes in Nebraska (Park et al. 2017), cranberry bogs in the northeast US (Loose et al. 2005) and wet flatwoods in Louisiana (Bartholomew and Prowell 2006). These studies and others have demonstrated that the vast majority of bee species found in wetlands also occur in terrestrial habitats and are therefore opportunistic wetland species. In fact, many bees found foraging in wetlands may nest in adjacent terrestrial habitats or in parts of the wetland complex that would not be delineated as, or considered, wetlands based on current definitions (Rust 1980,Cowardin et al. 1979). Nevertheless, a small number of bee species in North America are largely or entirely dependent on wetland communities, either because they depend on the pollen of certain wetland-obligate plants (e.g. *Ptilothrix bombiformis* depends on *Hibiscus* spp. pollen) or have certain nesting or development requirements other than pollen that may be provided only by wetlands, such as certain algae, which may play a role in providing oxygen to soil-nesting immature bees in seasonally flooded sites (Norden et al. 2003).

Reported here is the bee species list from the two-year study monitoring bee communities initiated in 2015 throughout the lower Mississippi Alluvial Valley (LMAV), an area dominated by agriculture and isolated wetlands. Our project represents the only work reported in the emergent wetlands of the LMAV, a region thought to have impaired bee species richness (Koh et al. 2015). Some properties surveyed are managed by the U.S. Fish and Wildlife Service, the Arkansas Game and Fish Commission, the Arkansas Natural Heritage Commission, while some are privately owned and managed. The purpose of our study was to compile an inventory of the bee fauna of emergent wetlands in the LMAV of Arkansas.

## Materials and methods

### Study Site

**Figure 1.**
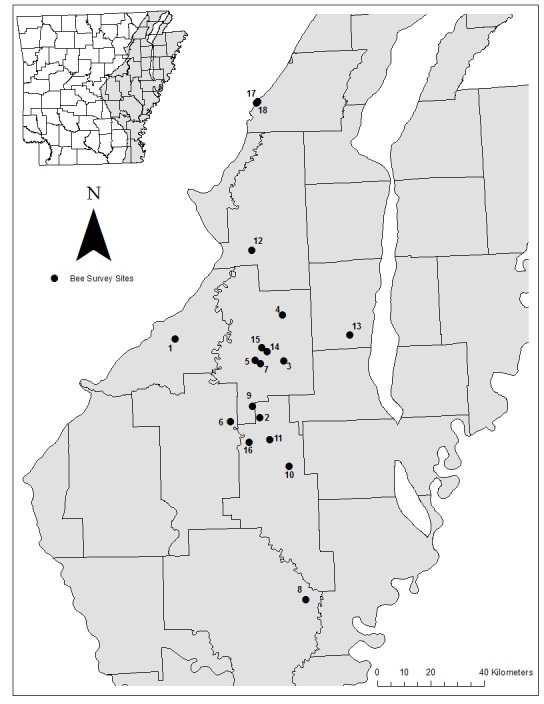
Distribution of managed palustrine emergent wetlands surveyed for bees in the lower Mississippi Alluvial Valley of Eastern Arkansas, USA in 2015 and 2016. See Table 1 for site names and coordinates.

The LMAV in Arkansas is bounded on the southwest by the West Gulf Coastal Plain and Ouachita Mountains, on the northwest by the Ozark Plateau and, on the east, by the Mississippi River. The elevation of the LMAV varies by only 46 m throughout the entire 402 km length of the LMAV in Arkansas (Crow 1974). The region is now dominated by agriculture (61% coverage; soybean, rice, corn, sorghum and cotton) with fragments of remnant emergent wetland (1%) and bottomland hardwood forest (17%) (King et al. 2006, USDA National Agricultural Statistics Service (NASS) 2016). The LMAV averages 118-134 cm of rainfall annually, with an average of 35 cm of rainfall between June and September (Scott et al. 1998). All of the 18 sites surveyed (Fig. 1) were used for agricultural or aquacultural production in the past 20 years before being restored to emergent wetlands. All sites had been impounded and were either being managed as moist-soil units, re-established to functioning emergent wetlands through the Wetland Reserve Program (WRP) or were naturally returning to emergent wetlands (Table 1). Wetlands ranged in size from 1 hectare to 50.5 hectares and periodically had standing water based on natural hydrology or water control structures.

Palustrine emergent wetlands are classified as areas <8 ha in size, lacking active wave-formed or bedrock shoreline features, water depth in the deepest part of the basin <2.5 m at low water and salinity due to ocean-derived salts less than 0.5 ppt (Cowardin et al. 1979). This wetland type is sometimes managed using soil disturbance (disking) or water level manipulation (control structures) to produce persistent or non-persistent vegetation for migratory birds (Fredrickson 1991). Persistent vegetation will remain erect when inundated with water and usually include rushes (*Juncus* spp.), cattails (*Typha* spp.), marsh-mallow (*Hibiscus* spp.) and perennial smartweeds (*Persicaria* spp.), while non-persistent vegetation will break over at the water line when inundated with water and usually include grasses (Poaceae), forbs (Asteraceae) and annual smartweeds (*Persicaria* spp.) (Cowardin et al. 1979: 41).

### Collection Methods

We captured bees by placing 10 pan trap stations approximately 20 m apart throughout managed emergent wetlands along a permanent transect following an opportunistic path avoiding open water. Pan trap station platforms held 3,266 ml Solo brand cups that were painted fluorescent blue, fluorescent yellow, or white (Droege et al. 2010, Kirk 1984, Leong and Thorp 1999). These cups were filled ¾ full with soapy water. Pan trap station platforms were adjusted to the average vegetation height at every collection point. We placed traps out at all sites between 0700-0900 hrs and collected them the same day between 1800-2000 hrs. We strained pan trapped bees using a 180 μm sieve in the field and transferred them to Whirl-Pak bags with 70% ethanol. We used one blue-vane trap (1.89 l jar) per field site suspended from a shepherds hook pole, with the bottom of the trap 1 m above the ground (Kimoto et al. 2012, Stephen and Rao 2005). The blue-vane trap was filled with 475 ml of soapy water. These blue-vane traps were placed and collected on the same schedule as the pan traps and samples similarly extracted. We used indirect sweep netting to sample for bees that were not attracted to either pan or blue-vane traps. We conducted 5 random transects of 50 sweeps apiece within each wetland per collection period to capture bees. Sweeps were conducted between 1100-1345 hrs (Stephen and Rao 2007) in 2015 and between 0900-1000 hrs (Roulston et al. 2007) in 2016. Sweep net collection periods were altered between years because we observed bees were more active between 0730-1000 hrs during the previous year. All sweep net samples were placed in 3.8 l Ziploc bags and were placed in the freezer until processed. We sampled each site 4-7 times in 2015 (19 May-18 September) and 8 times each in 2016 (22 May-9 September).

### Species Identification

Bee specimens were washed, dried, pinned and labelled with location information (Stephenson 2017). We identified bees to species when possible or to genus using identification guides and DiscoverLife.org (Ascher and Pickering 2017). We confirmed identifications with Harold Ikerd, Katherine Parys, Sam Droege and John Ascher. Voucher specimens are deposited at the University of Arkansas Arthropod Museum, Fayetteville, AR and at the U.S. National Pollinating Insect Collection, Logan, UT USA.

### Range

Species ranges and state records were determined using primary literature and other published accounts (see Literature Cited, below), the North American bee database available at DiscoverLife.org (Ascher and Pickering 2017) and, in a few cases, the bugguide website (bugguide.net).

### Wetland Affiliation

We classified bee species as “opportunistic,” “wetland-dependent," or "alluvial plain-restricted” based on published accounts and the ongoing surveys of one of the authors (MSA) in selected National Wildlife Refuges on the alluvial plains of the upper and middle Mississippi, lower Missouri and lower Ohio Rivers. The wetland-dependent and alluvial plain-restricted species are indicated by asterisks in the checklist below.

## Checklists

### Checklist

#### Colletidae

#### Colletes nudus

Robertson, 1898

##### Notes

Widespread east of the Rocky Mountains but not previously recorded from Arkansas (Stephen 1954). Opportunistic (Table 1: Site 15).

#### Hylaeus (Hylaeus) mesillae

(Cockerell, 1896)

##### Notes

Transcontinental but not previously recorded from Arkansas (Snelling 1970). Opportunistic (Table 1: Site 6).

#### Hylaeus (Prosopis) affinis

(Smith, 1853)

##### Notes

Widespread but not previously recorded from Arkansas (Hurd 1979). Opportunistic (Table 1: Site 1-3, 6, 14,17, 18).

#### Hylaeus (Prosopis) nelumbonis

(Robertson, 1890)

##### Notes

New record for Arkansas; previously recorded from Illinois and Maryland south to Florida and Louisiana (Mitchell 1960, Hurd 1979). Wetland specialist (Table 1: Sites 1-7,10, 12, 14, 15).

#### Hylaeus (Prosopis) ornatus

Mitchell, 1951

##### Notes

New record for Arkansas; previously recorded from North Carolina and Florida (Mitchell 1960, Hurd 1979). Wetland specialist (Table 1: Site 4, 5, 14, 15, 17).

#### Hylaeus sp. 1

##### Notes

(Table 1: Site 1).

#### Hylaeus sp. 2

##### Notes

(Table 1: Site 18).

#### Andrenidae

#### Andrena (Callandrena s.l.) rudbeckiae

Robertson, 1891

##### Notes

Known from the Great Plains east to North Carolina but not previously recorded from Arkansas (LaBerge 1967). Opportunistic (Table 1: Sites 5, 8).

#### Andrena (Leucandrena) macra

Mitchell, 1951

##### Notes

Known from the southeast to Texas but not previously recorded from Arkansas (LaBerge 1987). Opportunistic (Table 1: Sites 5, 7, 11, 14, 15, 17, 18).

#### Andrena (Scrapteropsis) imitatrix

Cresson, 1872

##### Notes

Opportunistic (Table 1: Site 5).

#### Andrena (Simandrena) nasonii

Robertson, 1895

##### Notes

Widespread in eastern North America west to Colorado and Texas but not previously recorded from Arkansas (LaBerge 1989). Opportunistic (Table 1: Site 17).

#### Calliopsis (Calliopsima) coloradensis

Cresson, 1878

##### Notes

Opportunistic (Table 1: Sites 3, 17, 18).

#### Panurginus polytrichus

Cockerell, 1909

##### Notes

Known from the adjacent states of Mississippi, Louisiana, and Texas but not previously recorded from Arkansas (Hurd 1979). Opportunistic (Table 1: Site 5).

#### Perdita (Hexaperdita) foveata

Timberlake, 1956

##### Notes

Opportunistic (Table 1: Site 4).

#### Perdita sp. 1

##### Notes

(Table 1: Sites 2, 7, 12, 15, 16).

#### Halictidae

#### Agapostemon angelicus/texanus

##### Notes

These specimens are most likely *A. texanus*, as the closet records to Arkansas of the predominantly western *A. angelicus* are from SE Oklahoma, while there are a number of *A. texanus* records from Arkansas (Roberts 1972). Opportunistic (Table 1: Sites 1-9, 11-18).

#### Agapostemon (Agapostemon) sericeus

(Forster, 1771)

##### Notes

Opportunistic (Table 1: Sites 1-15, 17-18).

#### Agapostemon (Agapostemon) splendens

(Lepeletier, 1841)

##### Notes

Opportunistic (Table 1: Sites 4-6, 13).

#### Agapostemon (Agapostemon) virescens

(Fabricius, 1775)

##### Notes

Opportunistic (Table 1: Sites 1,2, 4, 5, 7-12, 17).

#### Augochlora (Augochlora) pura

(Say, 1837)

##### Notes

Opportunistic (Table 1: Sites 5, 18).

#### Augochlorella aurata

(Smith, 1853)

##### Notes

Opportunistic (Table 1: All Sites).

#### Augochloropsis (Paraugochloropsis) fulgida

(Smith, 1853)

##### Notes

*Augochloropsis fulgida* and *A. metallica* (below) are here recognised as separate species, rather than subspecies as this has been the traditional interpretation (Moure and Hurd 1987). Studies by one of us (MSA) indicate that the two are largely sympatric and their distinguishing morphological features stable; molecular data appear to support this (S. Droege, in litt.). A short key separating the two is available at DiscoverLife.org, under *Augochloropsis metallica*. Opportunistic (Table 1: Sites 1, 11, 15, 18).

#### Augochloropsis (Paraugochloropsis) metallica

(Fabricius, 1793)

##### Notes

Opportunistic (Table 1: Sites 1-3, 5, 7, 8, 11, 12, 14).

#### Dieunomia (Epinomia) triangulifera

(Vachal, 1897)

##### Notes

New species record for Arkansas. Common in the central US usually on the alluvial plains of major rivers (Missouri, Arkansas) and their tributaries, east to the Mississippi River corridor and its tributaries in Missouri, Illinois and Indiana, but not recorded any further south along the Mississippi corridor until now (Cross 1958). A primary oligolege of *Helianthus* spp. and an important pollinator of *Helianthus annuus* and commercial sunflowers (*Minckley et al. 1994*). Primarily associated with alluvial plains of large rivers, not wetlands per se (Table 1: Site 8).

#### Halictus (Nealictus) parallelus

Say, 1837

##### Notes

Opportunistic (Table 1: All Sites).

#### Halictus (Odontalictus) ligatus

Say, 1837

##### Notes

Opportunistic (Table 1: All Sites).

#### Halictus (Protohalictus) rubicundus

(Christ, 1791)

##### Notes

This appears to be the first published Arkansas record of this common, widespread Holarctic species (Moure and Hurd 1987). Opportunistic (Table 1: Sites 1, 4, 9, 15, 18).

#### Lasioglossum (Dialictus) bruneri

(Crawford, 1902)

##### Notes

New species record for Arkansas; widespread in the eastern US (Gibbs 2011). Opportunistic (Table 1: Sites 1-3, 8, 9, 15).

#### Lasioglossum (Dialictus) callidum

(Sandhouse, 1924)

##### Notes

New species record for Arkansas; widespread in the eastern US (Gibbs 2011). Opportunistic (Table 1: Site 4).

#### Lasioglossum (Dialictus) creberrimum

(Smith, 1853)

##### Notes

New species record for Arkansas. *L. creberrimum* is a southeastern species, occurring largely along the coast from southeas Texas up to Maryland, with scattered inland records (Gibbs 2011). The Arkansas specimens represent the furthest inland occurrence of this species to date. Opportunistic (Table 1: All Sites).

#### Lasioglossum (Dialictus) cressonii

(Robertson 1890)

##### Notes

New species record for Arkansas; these specimens may represent the southernmost records for this common and widespread species (Gibbs 2011). Opportunistic (Table 1: Sites 1, 2, 4, 5, 7, 9, 12, 15).

#### Lasioglossum (Dialictus) hartii

(Robertson, 1892)

##### Notes

Restricted to alluvial plains and riparian corridors (Table 1: All Sites).

#### Lasioglossum (Dialictus) hitchensi

Gibbs, 2012

##### Notes

New species record for Arkansas. Widespread in the eastern US (Gibbs 2011, as *L. mitchelli* Gibbs 2010). Opportunistic (Table 1: Site 13).

#### Lasioglossum (Dialictus) pilosum

(Smith, 1853)

##### Notes

New species record for Arkansas. Occurs over much of the eastern US (Gibbs 2011). Opportunistic (Table 1: All Sites).

#### Lasioglossum (Dialictus) sp. 1

##### Notes

(Table 1: Sites 9, 13, 15, 16).

#### Lasioglossum (Dialictus) sp. 2

##### Notes

(Table 1: Sites 2, 10).

#### Lasioglossum (Hemihalictus) lustrans

(Cockerell, 1897)

##### Notes

Opportunistic (Table 1, Sites 2, 3, 5, 7, 9, 15, 18).

#### Lasioglossum (Hemihalictus) nelumbonis

(Robertson, 1890)

##### Notes

New species record for Arkansas. Occurs throughout much of the eastern US (Gibbs et al. 2013); primarily associated with alluvial plains and wetlands, but does occur in upland wetlands, upland pond margins, riparian areas etc (Table 1: All Sites).

#### Lasioglossum sp. 3

##### Notes

(Table 1: Sites 5, 9).

#### Nomia (Acunomia) nortoni

Cresson, 1868

##### Notes

Opportunistic (Table 1: Sites 5, 12-14).

#### Sphecodes mandibularis

Cresson, 1872

##### Notes

Opportunistic (Table 1: Site 5).

#### Megachilidae

#### Dianthidium (Dianthidium) subrufulum

Timberlake, 1943

##### Notes

Opportunistic (Table 1: Site 18).

#### Megachile (Acentron) albitarsis

Cresson, 1872

##### Notes

Widespread in eastern United States to Arizona but previously unrecorded from Arkansas (Mitchell 1937b). Opportunistic (Table 1: Sites 2, 3, 5,7, 8, 10, 14, 15, 17).

#### Megachile (Chelostomoides) campanulae

(Robertson, 1903)

##### Notes

Widespread in eastern United States west into Great Plains but previously unrecorded from Arkansas (Mitchell 1937a, Snelling 1990). Opportunistic (Table 1: Site 13).

#### Megachile (Leptorachis) petulans

Cresson, 1878

##### Notes

Widespread in eastern United States to Arizona but previously unrecorded from Arkansas (Mitchell 1937b). Opportunistic (Table 1: Sites 5, 7, 14, 17, 18).

#### Megachile (Litomegachile) brevis

Say, 1837

##### Notes

Widespread but previously unrecorded for Arkansas (Mitchell 1935, Bzdyk 2012). Opportunistic (Table 1: Sites 3-13, 15-18).

#### Megachile (Litomegachile) mendica

Cresson, 1878

##### Notes

Opportunistic (Table 1: Sites 1, 2, 10, 13-15).

#### Megachile (Litomegachile) texana

Cresson, 1878

##### Notes

Widespread but previously unrecorded for Arkansas (Mitchell 1935, Bzdyk 2012). Opportunistic (Table 1: Sites 2, 3, 15).

#### Apidae

#### Anthophorula (Anthophorisca) asteris

(Mitchell, 1962)

##### Notes

New species record for Arkansas; a fairly widespread (Texas to Georgia to Indiana) but infrequently-collected species (Ascher and Pickering 2017, Timberlake 1980). Opportunistic (Table 1: Site 15).

#### Apis (Apis) mellifera

Linnaeus, 1758

##### Notes

Opportunistic (Table 1: All Sites).

#### Bombus (Cullumanobombus) fraternus

(Smith, 1854)

##### Notes

Opportunistic (Table 1: Sites 5, 6, 14).

#### Bombus (Cullumanobombus) griseocollis

(De Geer, 1773)

##### Notes

Opportunistic (Table 1: Sites 1, 4, 8, 15).

#### Bombus (Pyrobombus) bimaculatus

Cresson, 1863

##### Notes

Opportunistic (Table 1: Sites 3, 8, 14, 15).

#### Bombus (Pyrobombus) impatiens

Cresson, 1863

##### Notes

Opportunistic (Table 1: Sites 3, 4, 13-15, 18).

#### Bombus (Thoracobombus) pensylvanicus

(De Geer, 1773)

##### Notes

Opportunistic Table 1: Sites 1, 2, 5-12, 16, 17).

#### Ceratina (Ceratinula) cockerelli

H.S. Smith, 1907

##### Notes

New species record for Arkansas; a southern and southeastern species, occurring from Texas to South Carolina (Daly 1973). Opportunistic (Table 1: Sites 7, 16).

#### Ceratina (Zadontomerus) dupla

Say, 1837

##### Notes

New species record for Arkansas, based on a male specimen. Common throughout much of the eastern half of the US (Daly 1973, Rehan and Sheffield 2011). Opportunistic (Table 1: Site 2).

#### Ceratina (Zadontomerus) sp. 1

##### Notes

(Table 1: Sites 1-3, 9, 12, 14, 15, 18).

#### Ceratina sp. 2

##### Notes

(Table 1: Site 2).

#### Diadasia (Diadasia) enavata

(Cresson, 1872)

##### Notes

New species record for Arkansas. This Asteraceae specialist occurs throughout most of the western half of the US; our specimens represent the easternmost location of the species published to date (Hurd et al. 1980), but they have been collected in some parts of Mississippi (Dr. Katherine Parys 2018, pers. comm., 8 February). Opportunistic (Table 1: Sites 5, 7-11, 15-18).

#### Eucera (Synhalonia) hamata

(Bradley, 1942)

##### Notes

New species record for Arkansas. Occurs throughout much of the eastern two-thirds of the US, but is absent from the states south and west of Arkansas (Hurd 1979, Timberlake 1969). Opportunistic (Table 1: Sites 2, 5, 7-9).

#### Eucera (Synhalonia) rosae

(Robertson, 1900)

##### Notes

Opportunistic (Table 1: Sites 2, 3, 10, 12, 13, 17, 18).

#### Florilegus (Florilegus) condignus

(Cresson, 1878)

##### Notes

New species record for Arkansas. A very widespread species, occurring throughout much of the eastern two-thirds of the US into Mexico, Central America and well into South America. No other native North American bee species has a similar or as extensive range. Populations in eastern North America are strongly associated with pickerelweed (*Pontedaria cordata* L.), alluvial plains and natural and constructed wetlands, including upland wetlands. The mouthparts of this species are festooned with hooked hairs (as are the mouthparts of the pickerelweed oligolege *Melissodes apicata* Robertson). *Florilegus condignus* females collect pollen from pickerelweed with their mouthparts as they hover, quickly “stabbing” the mouthparts in and out of the corolla. However, this species is not a strict oligolege of pickerelweed, as it also occurs in wetlands etc. where pickerelweed is absent. LaBerge and Ribble (1966) report western populations of this species to be potentially important pollinators of alfalfa. Primarily a wetland-dependent species in the eastern US portion of its range (Table 1: All Sites).

#### Melissodes (Eumelissodes) agilis

Cresson, 1878

##### Notes

New species record for Arkansas (LaBerge 1961). Opportunistic (Table 1: Sites 6, 7, 9).

#### Melissodes (Eumelissodes) boltoniae

Robertson, 1905

##### Notes

Opportunistic (Table 1: Sites 1, 4, 5, 7, 12-16, 18).

#### Melissodes (Eumelissodes) denticulatus

Smith, 1854

##### Notes

Opportunistic (: Site 17).

#### Melissodes (Eumelissodes) druriellus

(Kirby, 1802)

##### Notes

New species record for Arkansas (LaBerge 1956). Opportunistic (Table 1: Sites 1, 3, 4, 7, 11-15, 18).

#### Melissodes (Eumelissodes) niveus

Robertson, 1895

##### Notes

Opportunistic (Table 1: Site 5).

#### Melissodes (Eumelissodes) trinodis

Robertson, 1901

##### Notes

Opportunistic (Table 1: Site 7, 14, 15).

#### Melissodes (Melissodes) bimaculatus

(Lepeletier, 1825)

##### Notes

Opportunistic (Table 1: All Sites).

#### Melissodes (Melissodes) communis

Cresson, 1878

##### Notes

Opportunistic (Table 1: All Sites).

#### Melissodes (Melissodes) comptoides

Robertson, 1898

##### Notes

Opportunistic (Table 1: All Sites).

#### Melissodes (Melissodes) tepaneca

Cresson, 1878

##### Notes

Opportunistic (Table 1: Site 5, 8, 10, 11, 13, 15, 16).

#### Melitoma taurea

(Say, 1837)

##### Notes

Specimens from our study are the only Arkansas specimens we are aware of, but an Arkansas (Newton Co.) image of this species has recently been identified by JSA on bugguide.net, see https://bugguide.net/node/view/1259116. Opportunistic (Table 1: Sites 1, 3-6, 10-13, 15, 17).

#### Ptilothrix bombiformis

(Cresson, 1878)

##### Notes

Specimens from our study are the only Arkansas specimens we are aware of, but an Arkansas (Poinsett Co.) image of this species has recently been identified by JSA on bugguide.net, see https://bugguide.net/node/view/1422145. A wetlands specialist and Hibiscus oligolege (Rust 1980), but is occasionally found in developed areas some distance from wetlands visiting flowers of ornamental Hibiscus, or cultivated okra (Rau 1930). (Table 1: All Sites).

#### Svastra (Brachymelissodes) cressonii

(Dalla Torre, 1896)

##### Notes

New species record for Arkansas (LaBerge 1956). Opportunistic (Table 1: Sites 1, 5-7, 10, 13).

#### Svastra (Epimelissodes) atripes

(Cresson, 1872)

##### Notes

Opportunistic (Table 1: Sites 1-7, 9, 12-15, 17, 18).

#### Svastra (Epimelissodes) obliqua

(Say, 1837)

##### Notes

Opportunistic (Table 1: Sites 1-10, 12-18).

#### Svastra (Epimelissodes) petulca

(Cresson, 1878)

##### Notes

Opportunistic (Table 1: Sites 9, 16).

#### Triepeolus quadrifasciatus

(Say, 1823)

##### Notes

The type specimen, which is presumed lost or destroyed, was described from “Arcansa” in 1823 by Thomas Say (Rightmyer 2008). However, the “Arcansas” (Arkansas) of that era was a much larger piece of real estate than the Arkansas of today, then including most of what is now Oklahoma. No additional specimens from Arkansas are mentioned in Rightmyer (2008), thus we consider this specimen the first documentation of the species in Arkansas. Primary host bee is *Svastra atripes*. Opportunistic (Table 1: Site 1).

#### Xenoglossa (Eoxenoglossa) strenua

(Cresson, 1878)

##### Notes

New species record for Arkansas (Hurd and Linsley 1967). Opportunistic (Table 1: Sites 2, 6).

#### Xylocopa (Xylocopoides) virginica

(Linnaeus, 1771)

##### Notes

Opportunistic (Table 1: Sites 2-4, 8, 9, 11, 12, 14-16, 18).

## Analysis

During 201 collection events, between 2015 and 2016, we collected 17,860 bees representing 83 species and morphospecies across 31 genera and five families. Thirty-seven species captured represent new Arkansas state records.

## Discussion

Our study expands the known distribution of several of the bee species collected because of the limited documentation in emergent wetlands and especially for the LMAV. Our species list is relevant to other emergent wetlands in the LMAV, but may not reflect bee species in other ecoregions in Arkansas, especially in urban and upland areas along the Arkansas River Valley (see Little 2013). Most of the species collected are widespread in North America and many have been recorded from states that border Arkansas. While their presence in the state may not be surprising, the fact they have not been recorded highlights the lack of published data and surveys performed in this physiographic region and for this state.

### *Ceratina cockerelli*

*Ceratina cockerelli* is commonly associated with the Gulf Coastal Plains and the lower Piedmont ecoregions, but has been recorded outside of these ecoregions in West Texas. This species is the smallest *Ceratina* in eastern North America and is a generalist often associated with coastal habitats. The specimens collected represent a new state record for Arkansas and have expanded the known range of this species >200 km north into the Mississippi Alluvial Plain of Arkansas from its closest record in southwest Mississippi. These specimens were collected in Monroe and Woodruff Counties, Arkansas (Table 1: Sites 7, 16).

### *Diadasia enavata*

*Diadasia enavata* is commonly found in the western portion of the United States of America. This species is known to be restricted to plants in the Asteraceae family (Hurd et al. 1980, Linsley and MacSwain 1958). The wetlands surveyed occasionally have *Coreopsis tinctoria* on their edges being visited frequently by *Diadasia enavata*. *Coreopsis tinctoria* is found throughout the continental United States of America often in bottomland areas (USDA, NRCS 2017). The specimens collected represent a new state record for Arkansas and have considerably expanded the known range of this species east into the Mississippi Alluvial Plain of Arkansas from its closest records in Missouri, Oklahoma and Texas (Hurd et al. 1980). These specimens were collected in Arkansas, Lawrence, Monroe and Woodruff Counties, Arkansas (Table 1: Sites 5, 7-11, 15-18).

### *Dieunomia triangulifera*

*Dieunomia triangulifera* is a specialist of the sunflower genus *Helianthus* and is mainly found west of the Mississippi River and in the Great Plains of the United States of America (Cross 1958, Minckley et al. 1994). The wetlands surveyed had *Coreopsis tinctoria* present in the unit and on the levee. This specimen, collected in Arkansas County, represents a new state record for Arkansas (Table 1: Site 8).

### *Lasioglossum creberrimum*

*Lasioglossum creberrimum* is commonly associated with the Gulf Coastal Plains and Piedmont ecoregions of the United States of America, but has been recorded outside of these ecoregions in rare cases. This species is considered a generalist and prefers open lands. *Lasioglossum creberrimum* was also collected in remnant prairies and an urban park in the Arkansas River Valley in 2011-2012 (Little 2013). The specimens collected in the present study represent a new state record for Arkansas that expands the known range of this species >200 km north into the Mississippi Alluvial Plain of Arkansas from its closest record in southwest Mississippi. These specimens were collected in Arkansas, Cross, Jackson, Lawrence, Monroe, Prairie, White and Woodruff Counties, Arkansas (Table 1: All Sites).

### *Svastra cressonii*

*Svastra cressonii* is a species in the subgenus Brachymelissodes that is commonly found in the plains states of the south-central portion of the United States of America. This species has been collected as far north as Iowa (LaBerge 1956) and as far south as Louisiana (Ascher and Pickering 2017). The floral preferences of this species are unclear, but *Asteraceae* sp. and *Ludwigia peploides* have been mentioned (Estes and Thorp 1974, Ascher and Pickering 2017). Estes and Thorp (1974) documented *Svastra cressonii* foraging on *Ludwigia peploides* on the edges of farm ponds in Texas. This species of *Ludwigia* was present at all sites surveyed in our study. The collected *Svastra cressonii* specimens have expanded the known range >250 km east of previous documented occurrences. The specimens collected represent a new state record for Arkansas and expand the known range of this species into the Mississippi Alluvial Plain. These specimens were collected in Cross, Monroe, Prairie, White and Woodruff Counties, Arkansas (Table 1: Sites 1, 5-7, 10, 11, 13).

## Supplementary Material

XML Treatment for Colletidae

XML Treatment for Colletes nudus

XML Treatment for Hylaeus (Hylaeus) mesillae

XML Treatment for Hylaeus (Prosopis) affinis

XML Treatment for Hylaeus (Prosopis) nelumbonis

XML Treatment for Hylaeus (Prosopis) ornatus

XML Treatment for Hylaeus sp. 1

XML Treatment for Hylaeus sp. 2

XML Treatment for Andrenidae

XML Treatment for Andrena (Callandrena s.l.) rudbeckiae

XML Treatment for Andrena (Leucandrena) macra

XML Treatment for Andrena (Scrapteropsis) imitatrix

XML Treatment for Andrena (Simandrena) nasonii

XML Treatment for Calliopsis (Calliopsima) coloradensis

XML Treatment for Panurginus polytrichus

XML Treatment for Perdita (Hexaperdita) foveata

XML Treatment for Perdita sp. 1

XML Treatment for Halictidae

XML Treatment for Agapostemon angelicus/texanus

XML Treatment for Agapostemon (Agapostemon) sericeus

XML Treatment for Agapostemon (Agapostemon) splendens

XML Treatment for Agapostemon (Agapostemon) virescens

XML Treatment for Augochlora (Augochlora) pura

XML Treatment for Augochlorella aurata

XML Treatment for Augochloropsis (Paraugochloropsis) fulgida

XML Treatment for Augochloropsis (Paraugochloropsis) metallica

XML Treatment for Dieunomia (Epinomia) triangulifera

XML Treatment for Halictus (Nealictus) parallelus

XML Treatment for Halictus (Odontalictus) ligatus

XML Treatment for Halictus (Protohalictus) rubicundus

XML Treatment for Lasioglossum (Dialictus) bruneri

XML Treatment for Lasioglossum (Dialictus) callidum

XML Treatment for Lasioglossum (Dialictus) creberrimum

XML Treatment for Lasioglossum (Dialictus) cressonii

XML Treatment for Lasioglossum (Dialictus) hartii

XML Treatment for Lasioglossum (Dialictus) hitchensi

XML Treatment for Lasioglossum (Dialictus) pilosum

XML Treatment for Lasioglossum (Dialictus) sp. 1

XML Treatment for Lasioglossum (Dialictus) sp. 2

XML Treatment for Lasioglossum (Hemihalictus) lustrans

XML Treatment for Lasioglossum (Hemihalictus) nelumbonis

XML Treatment for Lasioglossum sp. 3

XML Treatment for Nomia (Acunomia) nortoni

XML Treatment for Sphecodes mandibularis

XML Treatment for Megachilidae

XML Treatment for Dianthidium (Dianthidium) subrufulum

XML Treatment for Megachile (Acentron) albitarsis

XML Treatment for Megachile (Chelostomoides) campanulae

XML Treatment for Megachile (Leptorachis) petulans

XML Treatment for Megachile (Litomegachile) brevis

XML Treatment for Megachile (Litomegachile) mendica

XML Treatment for Megachile (Litomegachile) texana

XML Treatment for Apidae

XML Treatment for Anthophorula (Anthophorisca) asteris

XML Treatment for Apis (Apis) mellifera

XML Treatment for Bombus (Cullumanobombus) fraternus

XML Treatment for Bombus (Cullumanobombus) griseocollis

XML Treatment for Bombus (Pyrobombus) bimaculatus

XML Treatment for Bombus (Pyrobombus) impatiens

XML Treatment for Bombus (Thoracobombus) pensylvanicus

XML Treatment for Ceratina (Ceratinula) cockerelli

XML Treatment for Ceratina (Zadontomerus) dupla

XML Treatment for Ceratina (Zadontomerus) sp. 1

XML Treatment for Ceratina sp. 2

XML Treatment for Diadasia (Diadasia) enavata

XML Treatment for Eucera (Synhalonia) hamata

XML Treatment for Eucera (Synhalonia) rosae

XML Treatment for Florilegus (Florilegus) condignus

XML Treatment for Melissodes (Eumelissodes) agilis

XML Treatment for Melissodes (Eumelissodes) boltoniae

XML Treatment for Melissodes (Eumelissodes) denticulatus

XML Treatment for Melissodes (Eumelissodes) druriellus

XML Treatment for Melissodes (Eumelissodes) niveus

XML Treatment for Melissodes (Eumelissodes) trinodis

XML Treatment for Melissodes (Melissodes) bimaculatus

XML Treatment for Melissodes (Melissodes) communis

XML Treatment for Melissodes (Melissodes) comptoides

XML Treatment for Melissodes (Melissodes) tepaneca

XML Treatment for Melitoma taurea

XML Treatment for Ptilothrix bombiformis

XML Treatment for Svastra (Brachymelissodes) cressonii

XML Treatment for Svastra (Epimelissodes) atripes

XML Treatment for Svastra (Epimelissodes) obliqua

XML Treatment for Svastra (Epimelissodes) petulca

XML Treatment for Triepeolus quadrifasciatus

XML Treatment for Xenoglossa (Eoxenoglossa) strenua

XML Treatment for Xylocopa (Xylocopoides) virginica

## Figures and Tables

**Table 1.** Site number, site name, ownership, latitude, longitude, county, hectares and year surveyed during 2015 and 2016 in the lower Mississippi Alluvial Valley of Eastern Arkansas, USA.

Year Surveyed								
**Site Number**	**Study Site^a^**	**Ownership^b^**	**Latitude**	**Longitude**	**County**	**Hectares**	**2015**	**2016**
1	Bald Knob NWR	USFWS	35.210614	-91.608737	White	7.7	X	X
2	Benson Creek Natural Area WMA	ANHC	34.932789	-91.272666	Monroe	12	X	X
3	Cache River NWR Cabin	USFWS	35.118294	-91.160946	Woodruff	1.5	-	X
4	Cache River NWR Hwy 64	USFWS	35.273179	-91.156697	Woodruff	8.8	-	X
5	Cache River NWR Lower Howell Unit	USFWS	35.126017	-91.281515	Woodruff	6.9	X	X
6	Cache River NWR Plunkett Farm Unit	USFWS	34.92312	-91.395941	Prairie	50.5	X	-
7	Cache River NWR Upper Howell Unit	USFWS	35.112987	-91.259239	Woodruff	11.5	X	X
8	Dale Bumpers White River NWR Farm Pond #2	USFWS	34.311726	-91.121353	Arkansas	1	-	X
9	Gin Road	Private	34.971019	-91.302877	Woodruff	3.6	-	X
10	Gumbo 241	Private	34.764475	-91.161115	Monroe	4	X	X
11	Hallum Cemetery Road	Private	34.857014	-91.236786	Monroe	6.7	-	X
12	Jackson County Hwy 224	Private	35.495896	-91.273169	Jackson	4.3	-	X
13	Oldham Duck Club	Private	35.193993	-90.882663	Cross	4.7	-	X
14	Rex Hancock Black Swamp WMA Wiville Unit East	AGFC	35.153624	-91.228901	Woodruff	4.1	X	X
15	Rex Hancock Black Swamp WMA Wiville Unit West	AGFC	35.167774	-91.250383	Woodruff	3.2	X	X
16	Sheffield Nelson Dagmar WMA Conway George Unit C	AGFC	34.852126	-91.324203	Monroe	13.2	X	X
17	Shirey Bay Rainey Brake WMA North	AGFC	35.994752	-91.217169	Lawrence	6.9	X	X
18	Shirey Bay Rainey Brake WMA South	AGFC	35.988878	-91.221381	Lawrence	2.8	X	X
^a^NWR - National Wildlife Refuge, WMA - Wildlife Management Area, ^b^USFWS - US Fish and Wildlife Service, ANHC - Arkansas Natural Heritage Commission, Private - Private land, AGFC - Arkansas Game and Fish Commission								
